# Serum IL-33 level is associated with auto-antibodies but not with clinical response to biologic agents in rheumatoid arthritis

**DOI:** 10.1186/s13075-018-1628-6

**Published:** 2018-06-08

**Authors:** Elodie Rivière, Jérémie Sellam, Juliette Pascaud, Philippe Ravaud, Jacques-Eric Gottenberg, Xavier Mariette

**Affiliations:** 1 Immunology of viral Infections and Autoimmune Diseases, IDMIT, CEA - Université Paris Sud - INSERM UMR1184, Le Kremlin Bicêtre & Fontenay aux Roses, France; 2 Department of Rheumatology, Saint-Antoine Hospital, Université Paris 6, INSERM UMRS 938, DHU i2B, Paris, France; 3 Department of Epidemiology and Biostatistics, Hotel Dieu, Paris, France; 4 Department of Rheumatology, National Reference Center for Systemic Autoimmune Diseases, Strasbourg University Hospital, Université de Strasbourg, Strasbourg, France; 5 Department of Rheumatology, Université Paris Sud, 63 rue Gabriel Péri, 94270 Le Kremlin Bicetre, France

**Keywords:** Interleukin 33, Personalized medicine, Rheumatoid arthritis, Biologic agents

## Abstract

Rotation or Change of Biotherapy After First Anti-TNF Treatment Failure for Rheumatoid Arthritis (ROC), registered 22 October 2009, NCT01000441

Interleukin (IL)-33 may play a role in rheumatoid arthritis (RA) pathophysiology as shown by human studies and murine models [1]. Previously, we demonstrated that detectable serum IL-33 predicts clinical response to rituximab independently of auto-antibody status [2].

Here, we aimed to investigate whether the prediction of therapeutic response using serum IL-33 level is generalizable to all biologic agents, including TNF inhibitors (TNFi) and non-TNFi in RA.

We set up an ancillary study of the ROC (Rotation or Change of Biotherapy After First Anti-TNF Treatment Failure for RA) trial (NCT01000441) which compared the efficacy of TNFi vs non-TNFi in patients with insufficient response to a first TNFi [3]. Three hundred patients were randomized, and treatment efficacy was evaluated at 24 weeks according to EULAR response, showing that a non-TNFi was more effective in achieving EULAR response than a TNFi. Serum IL-33 level was assessed before treatment using an accurate enzyme-linked immunosorbent assay (ELISA IL-33, Quantikine, R&D Systems) [4]. Statistical analyses used Prism (Mann-Whitney and Fisher tests for quantitative and qualitative values, respectively). Serum IL-33 level was defined as detectable when > 6.25 pg/mL (lower threshold).

Results were analyzed for 267 patients with available serum and clinical data (Table 1). Serum IL-33 level was detectable for 109/267 (40.8%) patients (mean ± standard deviation serum level was 49.7 ± 61.0 pg/mL when detectable) (Table 2). IL-33 detection was associated with auto-antibody positivity: rheumatoid factor (RF) and/or anti-cyclic citrullinated peptide antibody (anti-CCP), either combined or analyzed separately (Table 3). Auto-antibody positivity was not associated with response to the different treatment: TNFi (*N* = 132, odds ratio (OR) = 1.1, 95% confidence interval (CI) = 0.39–3.16), non-TNFi (*N* = 130, OR = 1.5, 95% CI = 0.40–5.62), or different sub-groups of non-TNFi (data not shown). There was no association between IL-33 detection and response to TNFi as well as to non-TNFi drugs overall or analyzed separately (Table 2). Likewise, there was no difference when comparing the levels of serum IL-33 between responders and non-responders in TNFi and non-TNFi groups (data not shown).

**Table 1:** Characteristics of the patients included in the ancillary study of the ROC trial

Characteristics	TNFi	Non-TNFi biologic	Total
Number of women (%)	114 (85.7)	110 (82.1)	224 (83.9)
Mean age (SD)	55.9 (13.0)	58.4 (11.2)	57.2 (12.1)
Number rheumatoid factor-positive (%)	108 (82.4)	101 (76.5)	209 (79.8)
Number anti-CCP-positive (%)	102 (79.7)	105 (82.7)	207 (81.2)
Mean DAS28-CRP (SD)	4.7 (0.9)	4.8 (1.1)	4.8 (1.0)

**Table 2:** EULAR response, IL-33 detectability rates and association between IL-33 detection and response to tumor necrosis factor inhibitor (TNFi; including adalimumab, certolizumab, etanercept and infliximab) and non-TNFi (including abatacept, rituximab, and tocilizumab) in patients from the ROC study

	Treatment	Number of patients	Number of EULAR responders (%)	Number of detectable IL-33 among all patients (%)	Number of detectable IL-33 among EULAR responders (%)	Association between IL-33 detectability and EULAR response (OR [95% CI])
TNFi	Adalimumab	53	30 (56.6)	23 (43.4)	14 (46.7)	1.4 [0.5–4.1]
	Etanercept	49	29 (59.2)	20 (40.8)	13 (44.8)	1.5 [0.5–5.0]
	Certolizumab	23	10 (43.5)	10 (43.5)	5 (50.0)	1.6 [0.3–8.5]
	Infliximab	8	1 (12.5)	3 (37.5)	1 (100)	6.6 [0.2–226]
	Total TNFi	133	70 (52.6)	56 (42.1)	33 (47.1)	1.6 [0.8–3.1]
Non-TNFi	Rituximab	37	20 (54.0)	10 (27.0)	6 (30.0)	1.4 [0.3–6.1]
	Abatacept	30	18 (60.0)	11 (36.6)	8 (44.4)	2.4 [0.5–11.9]
	Tocilizumab	67	53 (79.1)	32 (47.8)	25 (47.2)	0.9 [0.3–2.9]
	Total non-TNFi	134	91 (67.9)	53 (39.6)	39 (42.9)	1.6 [0.7–3.3]

Results are presented as odds ratios (OR) [95% confidence intervals (CI)]

**Table 3:** Association between IL-33 detection and auto-antibody positivity

Auto-antibody status	Number of patients	OR	95% CI
RF+ and/or anti-CCP+ vs RF− and anti-CCP−	262	21.1	2.8-158.3
RF+ vs RF−	263	9.7	3.7-25.3
Anti-CCP+ vs anti-CCP−	255	2.7	1.3- 5.7

Results are presented as odds ratios (OR), 95% confidence intervals (CI) for each factor

*RF* rheumatoid factor, *Anti-CCP* anti-cyclic citrullinated peptide antibody

Thus, this new study confirms the association between serum IL-33 detection and seropositivity in RA patients. However, it did not replicate the association between IL-33 detection and response to rituximab. This may be due to a lack of power related to the number of patients who received this treatment (*N* = 37), but it may also reflect the difficulty of studying IL-33 as a possible predictor of response given its association with seropositivity, which is a well-known factor associated with response to some biologics such as rituximab or abatacept [5].

In conclusion, we confirm that serum IL-33 detection is associated with auto-antibody positivity but is not a predictive marker for response to TNFi and non-TNFi in RA.

## Abbreviations

Anti-CCP: Anti-cyclic citrullinated peptide
CI: Confidence interval
ELISA: Enzyme-linked immunosorbent assay
EULAR: European League Against Rheumatism
Ig: Immunoglobulin
IL: Interleukin
OR: Odds ratio
RA: Rheumatoid arthritis
RF: Rheumatoid factor
TNFi: Tumor necrosis factor inhibitor
